# Novel oncogenic function of *mesoderm development candidate 1* and its regulation by *MiR-574-3p* in bladder cancer cell lines

**DOI:** 10.3892/ijo.2011.1294

**Published:** 2011-12-13

**Authors:** SHUICHI TATARANO, TAKESHI CHIYOMARU, KAZUMORI KAWAKAMI, HIDEKI ENOKIDA, HIROFUMI YOSHINO, HIDEO HIDAKA, NIJIRO NOHATA, TAKESHI YAMASAKI, TAKENARI GOTANDA, TOKUSHI TACHIWADA, NAOHIKO SEKI, MASAYUKI NAKAGAWA

**Affiliations:** 1 Department of Urology, Graduate School of Medical and Dental Sciences, Kagoshima University, Kagoshima; 2 Department of Functional Genomics, Graduate School of Medicine, Chiba University, Chiba, Japan

**Keywords:** microRNA, *miR-574-3p*, *mesoderm development candidate 1*, bladder cancer

## Abstract

Our previous studies suggested that *microRNA* (*miR*)*-574-3p* is a candidate tumor suppressor microRNA (miRNA) in human bladder cancer (BC). Among 17 down-regulated miRNAs, *miR-574-3p* is located on chromosome 4p14 where we had identified a chromosomal loss region by array-CGH in BC cell lines. *MiR-574-3p* expression was down-regulated in BC cell lines. Gain-of-function analysis revealed that cell proliferation, migration and invasion were significantly inhibited in *miR*-*574*-*3p*-transfected BC cell lines. Flow cytometry analysis showed that cell apoptosis was induced in *miR-574-3p* transfectants. Oligo microarray analysis suggested that the *mesoderm development candidate 1* (*MESDC1*) gene was a target gene in *miR-574-3p* transfectants. Luciferase assays revealed that *miR*-*574*-*3p* was directly bound to *MESDC1* mRNA. MESDC1 is predicted to be a novel actin-binding protein located on chromosome 15q13. Although the gene is conserved among many species, its functional role is still unknown in both human malignancies and normal tissues. Loss-of-function studies demonstrated that cell proliferation, migration and invasion were significantly inhibited in si-MESDC1-transfected BC cell lines. Flow cytometry analysis showed that apoptosis was induced in si-MESDC1 transfectants. We are the first to demonstrate that *miR-574-3p* is a miRNA with tumor suppressor function and that MESDC1 (which has a potential oncogenic function in BC) may be targeted by *miR-574-3p*.

## Introduction

Bladder cancer (BC) is the fourth most common malignancy in economically developed nations (1). In Japan, there were 16,477 new cases in 2005 and 6,625 deaths in 2009 (2). There have been significant advances in treatment, including surgical techniques and adjuvant chemotherapy. However, BC frequently recurs and a poor clinical outcome is anticipated when the cancer progresses to muscle-invasive disease (3). It is crucial to investigate the mechanism of carcinogenesis and novel molecular target genes that have a tumor suppressive or oncogenic function in BC.

MicroRNAs (miRNAs) constitute a class of regulatory RNA with a length of 20–24 nucleotides, and they post-transcriptionally regulate gene expression in eukaryotes. Although their precise biology is not fully understood, miRNAs are found in diverse organisms and epigenetically function as negative regulators of gene expression. They are associated with cell growth, cell cycle control, invasion, proliferation, migration, and evasion of apoptosis in oncogenesis (4). They also play an important role in oncogenesis in BC and behave in an oncogenic or tumor suppressive manner in various cancers (5–7). To date, many reports have demonstrated significant roles of miRNAs in BC (8–13). We previously demonstrated that several down-regulated miRNAs, such as *miR-1, miR-133a, miR-145, miR-218* and *miR-517a*, have tumor suppressive function by targeting oncogenes. It is noteworthy that ‘actin binding protein’ was often included in the functional annotation of these miRNAs target genes, such as *fascin homologue 1* (*FSCN1*), *LIM* and *SH3 protein 1* (*LASP1*), and *transgelin-2* (*TAGLN2*) (14–16), and restoration of these miRNAs induced cell apoptosis (16–18).

In this study, we focused on *miR-574-3p*, which was selected from the 17 down-regulated miRNAs of BC in our previous study (16). *MiR-574-3p* as well as *miR-218*, a tumor suppressive miRNA (17), are located on chromosome 4p (4p14 and 4p15), which was a typical chromosomal loss region in BC cell lines in our previous study (18). We found that *miR*-*574-3p* was indeed down-regulated in BC cell lines. To find the target genes of *miR*-*574-3p*, we performed an oligo-microarray study of *miR*-*574-3p* transfectants. We found that *MESDC1* was the most down-regulated gene and has a putative target site for *miR*-*574-3p*. MESDC1 is predicted to be a novel actin-binding protein located on chromosome 15q13. Although the gene is conserved among many species, functional studies of MESDC1 are lacking in the literature. We hypothesized that *miR*-*574-3p* directly regulates *MESDC1* and that this gene has oncogenic activity through its anti-apoptotic function in BC. We performed a luciferase reporter assay to determine whether *MESDC1* mRNA was actually targeted by *miR-574-3p* and loss-of-function studies using BC cell lines to investigate functional roles of MESDC1 in BC.

## Materials and methods

### BC cell lines and cell culture

We used two human BC cell lines: BOY, which was established in our laboratory from an Asian male patient, age 66, and diagnosed with stage III BC with lung metastasis (20); T24 was obtained from the American Type Culture Collection. These cell lines were maintained in minimum essential medium (MEM) supplemented with 10% fetal bovine serum (FBS) in a humidified atmosphere of 5% CO_2_ and 95% air at 37°C.

### Tissue samples

Tissue samples were taken from 24 BC patients who had undergone cystectomy or transurethral resection of BCs at Kagoshima University Hospital between 2007 and 2009. The median age of the patients was 71 years, ranging from 62 to 88 years. The BC samples were from 14 non-muscle invasive (<T2) and 10 muscle invasive (≥T2) cancers; 10 were low grade BC and the other 14 were high grade BC. The samples were staged in accordance with the tumor-node-metastasis classification system of the American Joint Committee on Cancer-Union Internationale Contre le Cancer (UICC) and were histologically graded (21). The study was approved by the Bioethics Committee of Kagoshima University; written prior informed consent and approval were given by the patients.

### Tissue collection and RNA extraction

Tissue samples were immersed in RNAlater (Qiagen, Valencia, CA, USA) and stored at −20°C until RNA was extracted. Total RNA (including miRNA) was extracted from frozen fresh tissues using the mirVana™ miRNA isolation kit (Ambion, Austin, TX, USA) in accordance with the manufacturer’s protocol. The integrity of the RNA was checked with an RNA 6000 Nano Assay Kit and a 2100 Bioanalyzer™ (Agilent Technologies, Santa Clara, CA, USA).

### Quantitative real-time RT-PCR

TaqMan probes and primers for *MESDC1* (TaqMan^®^ Gene Expression Assays, P/N: Hs00739656_s1, Applied Biosystems, Foster City, CA, USA) were assay-on-demand gene expression products. All reactions were performed in duplicate, and a negative control lacking cDNA was included. We followed the manufacturer’s protocol for the PCR conditions. Stem-loop RT–PCR for *miR*-*574-3p* (TaqMan^®^ MicroRNA Assays, P/N: 002349, Applied Biosystems) was used to quantitate miRNAs according to the earlier published conditions (10). cDNA was made from 5 ng of total RNA from each sample using the TaqMan^®^ MicroRNA Reverse Transcription Kit (Applied Biosystems). For quantitative analysis of mRNA and miRNA, we used human *18s rRNA* (P/N: 4319413E, Applied Biosystems) and *RNU48* (P/N: 001006, Applied Biosystems) as an internal control, and we used the delta-delta Ct method to calculate the fold-change. As control RNA, we used three different lots of Premium Total RNA from normal human bladder (AM7990, Applied Biosystems).

### Mature miRNA and siRNA transfection

As described elsewhere (10), the BC cell lines were transfected with Lipofectamine™ RNAiMAX transfection reagent (Invitrogen, Carlsbad, CA, USA) and Opti-MEM™ (Invitrogen) with 10 nM of mature miRNA molecules. Mature miRNA molecules, Pre-miR™ (*hsa-miR-574-3p*, P/N: AM17100, Applied Biosystems) and negative control miRNA (P/N: AM17111, Applied Biosystems) were used in the gain-of-function experiments, whereas *MESDC1* siRNA (Cat# HSS126949 and HSS126950, Invitrogen) and negative control siRNA (D-001810-10, Thermo Fisher Scientific, Waltham, MA, USA) were used in the loss-of-function experiments. Cells were seeded in 10-cm dishes for protein extraction (8×10^5^ per dish), in 6-well plates for apoptosis assays (10×10^4^ per well) and for wound healing assays (20×10^4^ per well), in 24-well plates for mRNA extraction and luciferase reporter assays (5×10^4^ per well), and in 96-well plates for XTT assays (3,000 per well).

### Cell viability, migration, and invasion assays

Cell viability was determined by using an XTT assay (Roche Applied Sciences, Tokyo, Japan) performed according to the manufacturer’s instructions. Cell migration activity was evaluated by wound-healing assays. Cells were plated in 6-well dishes, and the cell monolayer was scraped using a P-20 micropipette tip. The initial gap length (0 h) and the residual gap length 24 h after wounding were calculated from photomicrographs. A cell invasion assay was carried out using modified Boyden Chambers consisting of transwell-precoated matrigel membrane filter inserts with 8 μm pores in 24-well tissue culture plates (BD Biosciences, Bedford, MA, USA). MEM containing 10% FBS in the lower chamber served as the chemo-attractant, as described previously (14). All experiments were performed in triplicate.

### Apoptosis analysis

BC cell lines were transiently transfected with transfection reagent only (mock), miR-control, *miR*-*574-3p*, si-control, or si-MESDC1 in 6-well tissue culture plates, as described earlier. Cells were harvested 72 h after transfection by trypsinization and washed in cold PBS. Double staining with FITC-Annexin V and propidium iodine (PI) was carried out using the FITC Annexin V Apoptosis Detection Kit (BD Biosciences) according to the manufacturer’s recommendations and analyzed within 1 h by flow cytometry (FACScan^®^, BD Biosciences). Cells were discriminated into viable cells, dead cells, early apoptotic cells and apoptotic cells by CellQuest software (BD Biosciences), and then the percentages of early apoptotic cells from each experiment were compared. Experiments were done in triplicate.

### Target gene search for miR-574-3p

Oligo-microarray human 44K (Agilent) was used for expression profiling of *miR*-*574-3p*-transfected BC cell lines (BOY and T24) in comparison with miR-negative control transfectants, as previously described (14). Briefly, hybridization and washing steps were performed in accordance with the manufacturer’s instructions. The arrays were scanned using a Packard GSI Lumonics ScanArray^®^ 4000 (Perkin-Elmer, Boston, MA, USA). The data obtained were analyzed with DNASIS^®^ array software (Hitachi Software Engineering, Tokyo, Japan), which converted the signal intensity for each spot into text format. The log2 ratios of the median subtracted background intensity were analyzed. Data from each microarray study were normalized by global normalization.

The predicted target genes and their miRNA binding site seed regions were investigated using TargetScan program (release 5.2, http://www.targetscan.org/), MicroRNA.org (released August 2010, http://www.microrna.org/) and Micro Cosm Targets (version 5, http://www.ebi.ac.uk/enright-srv/microcosm/htdocs/targets/v5/). The sequences of the predicted mature miRNAs were confirmed using miRBase (released 16.0, Sept 2010; http://microrna.sanger.ac.uk/).

### Plasmid construction and dual-luciferase reporter assay

MiRNA target sequences were inserted between the SgfI-PmeI restriction sites in the 3′UTR of the hRluc gene in the psiCHECK™-2 vector (C8021, Promega, Madison, WI, USA). Primer sequences for full-length 3′UTR of *MESDC1* mRNA (TACGCGATCGCGTCTTCGCCCAGGACTTTAC and TAGGTTTAAACAAACTTGACGTTGGGGGAAA) were designed. Following that, BOY and T24 cells were transfected with 15 ng of vector, 10 nM of miRNA, and 1 μl of Lipofectamine™ 2000 (Invitrogen) in 100 μl of Opti-MEM™ (Invitrogen). The activities of firefly and *Renilla* luciferases in cell lysates were determined with a dual-luciferase assay system (E1910; Promega). Normalized data were calculated as the quotient of *Renilla*/firefly luciferase activities.

### Statistical analysis

The relationship between two variables and the numerical values obtained by real-time RT-PCR was analyzed using the Mann-Whitney U test. The relationship between three variables and the numerical values was analyzed using the Bonferroni-adjusted Mann-Whitney U test. Expert StatView^®^ analysis software (version 4, SAS Institute Inc., Cary, NC, USA) was used in both cases. In the comparison of three variables, a non-adjusted statistical level of significance of P<0.05 corresponds to a Bonferroni-adjusted level of P<0.0167.

## Results

### Detection of miR-574-3p expression in clinical BCs and BC cell lines by quantitative stem-loop RT-PCR

**Figure 1:**
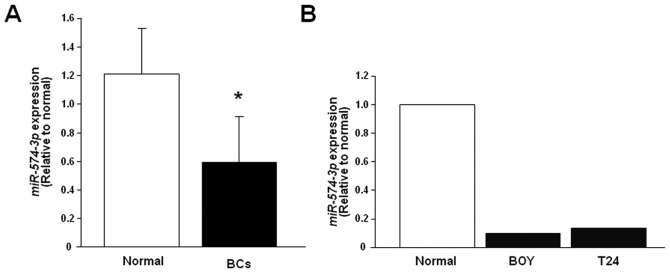
*MiR-574-3p* expression levels in (A) clinical BCs and (B) BC cell lines compared with the normal human bladder RNA. ^*^P<0.05.

Quantitative stem-loop RT-PCR demonstrated that the expression level of *miR-574-3p* was significantly lower in BCs (n=24) than in the normal human RNAs (P=0.0128, Fig. 1A) and that it was markedly lower in the BC cell lines (Fig. 1B). We found no significant correlations between *miR-574-3p* expressions and pathological parameters.

### Effect of miR-574-3p transfection on cell viability, migration, invasion activity, and cell apoptosis in BC cell lines

**Figure 2:**
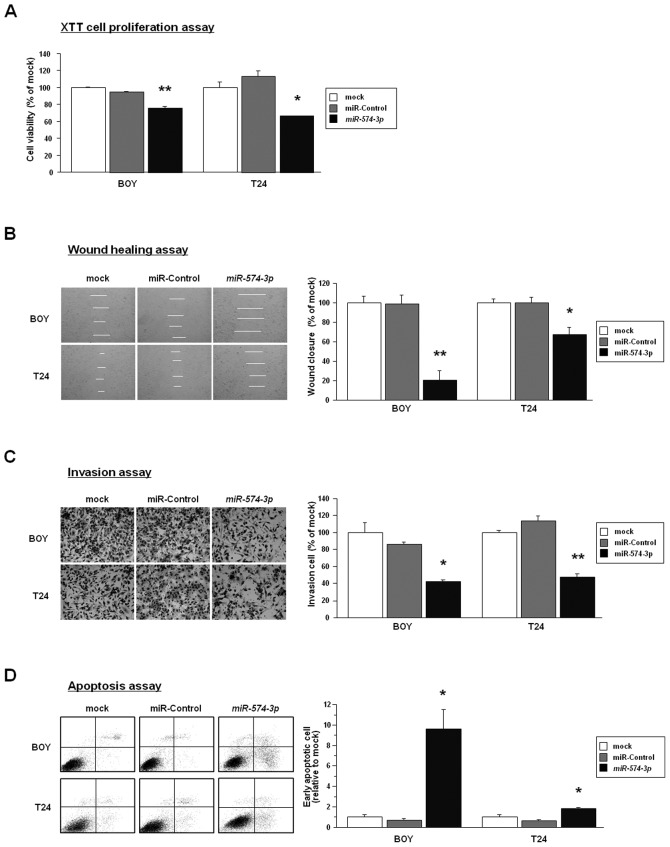
Gain-of-function studies in *miR-574-3p*-transfected BC cell lines. (A), Cell proliferation determined by the XTT assays of the transfectants. ^*^P<0.001. ^**^P<0.0001. (B), Wound healing assays demonstrated significant inhibition of cell migration in *miR-574-3p* transfectants. Phase contrast micrographs of the transfectants (BOY and T24) taken at 0 and 24 h after monolayer wounding are shown on the left. Quantification of cell migration using the monolayer wound healing assay is shown on the right. ^*^P<0.001. ^**^P<0.0001. (C), Significant inhibition of cell invasion was observed in *miR-574-3p* transfectants. Phase contrast micrographs of invading transfectants (BOY and T24) are shown on the left. Quantitation of cell invasion is shown in the right panel. ^*^P<0.01. ^**^P<0.0001. (D), Apoptosis assay by flow cytometry. Significant numbers of early apoptotic cells were observed in *miR-574-3p* transfectants. Early apoptotic cells can be seen in the bottom right quadrant. The percentages of early apoptotic cells in the miR-control and the *miR-574-3p* transfectants are shown in the histogram. ^*^P<0.01.

To investigate the functional role of *miR*-*574*-*3p*, we performed gain-of-function studies using *miR*-*574*-*3p*-transfected BC cell lines (BOY and T24). The XTT assay demonstrated that cell proliferation was reduced to 76% and 66% of mock in *miR*-*574*-*3p*-transfected BOY and T24 (P<0.0001 and P<0.0005, Fig. 2A). The wound healing assay demonstrated that wound closure ratio was reduced to 20% and 67% of mock in *miR*-*574*-*3p*-transfected BOY and T24 (P<0.0001 and P=0.0004, Fig. 2B). The Matrigel invasion assay demonstrated that cell invasion ratio was reduced to 42% and 48% of mock in *miR*-*574*-*3p*-transfected BOY and T24 (each P<0.0001, Fig. 2B). The early apoptotic cell fractions (right lower quadrant) were greater in the *miR-574-3p* transfectants than in the mocks and the miR-control transfectants (relative to mock; BOY: 9.60±1.91, 1.00±0.25, and 0.71±0.15, respectively, P=0.0013; T24: 1.84±0.08, 1.00±0.22, and 0.65±0.11, respectively, P=0.0014) (Fig. 2D). These results suggested that restoration of *miR-574-3p* expression might induce cell apoptosis in BC.

### Gene expression signatures of differentially down-regulated genes in miR-574-3p transfectants

To gain further insight into which genes were affected by *miR-574-3p* transfection, we performed gene expression analysis with *miR-574-3p* transfectants (BOY and T24). A total of 11 genes were down-regulated less than −2.0-fold in the *miR-574-3p* transfectants compared with the control transfectants (Table I). MicroRNA.org and MicroCosm Target programs showed the eight genes had putative target sites for *miR-574-3p* in their 3′UTR (Table I). The *MESDC1* gene was the most down-regulated gene that had conserved target sites for *miR-574-3p*. Therefore, we focused on *MESDC1* as a promising candidate targeted by *miR-574-3p*. Entries from the former and the current microarray data were approved by the Gene Expression Omnibus (GEO) and were assigned GEO accession number, GSE24782.

### MESDC1 expression in BC cell lines and MESDC1 silencing by miR-574-3p transfection

**Figure 3:**
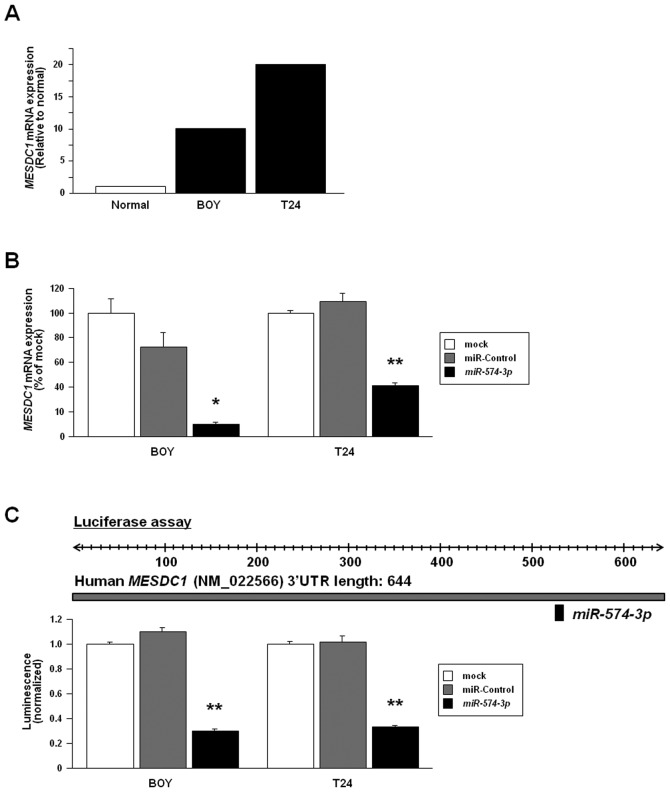
(A), *MESDC1* mRNA expression levels in BC cell lines compared with the normal human bladder RNA. (B), *MESDC1* mRNA expression was repressed in the *miR-574-3p* transfectants. ^*^P<0.01. ^**^P<0.0001. (C), Luciferase reporter assays using the vector encoding full-length 3′UTR of *MESDC1* mRNA. The *Renilla* luciferase values were normalized to firefly luciferase values. ^**^P<0.05.

The quantitative real-time RT-PCR analysis showed that mRNA expression levels of *MESDC1* in BOY and T24 were higher than that in the normal human bladder RNAs (Fig. 3A). In clinical BCs, there was no significant difference in *MESDC1* mRNA expression between clinical BCs and the normal human bladder RNAs (data not shown). We performed gain-of-function studies using *miR-574-3p*-transfected BOY and T24. The mRNA expression levels of *MESDC1* were markedly repressed in the *miR-574-3p* transfectants in comparison with the miR-control transfectants and the mocks (Fig. 3B).

### Confirmation of MESDC1 as a target of post-transcriptional repression by miR-574-3p

We performed a luciferase reporter assay to determine whether *MESDC1* mRNA has an actual target site for *miR-574-3p.* We used a vector encoding full-length 3′UTR of *MESDC1* mRNA and found that the luminescence intensity was significantly reduced in the *miR-574-3p* transfectants compared to their counterparts (Fig. 3C).

### Effect of MESDC1 knockdown on cell viability, migration, invasion activity, and cell apoptosis in BC cell lines

**Figure 4:**
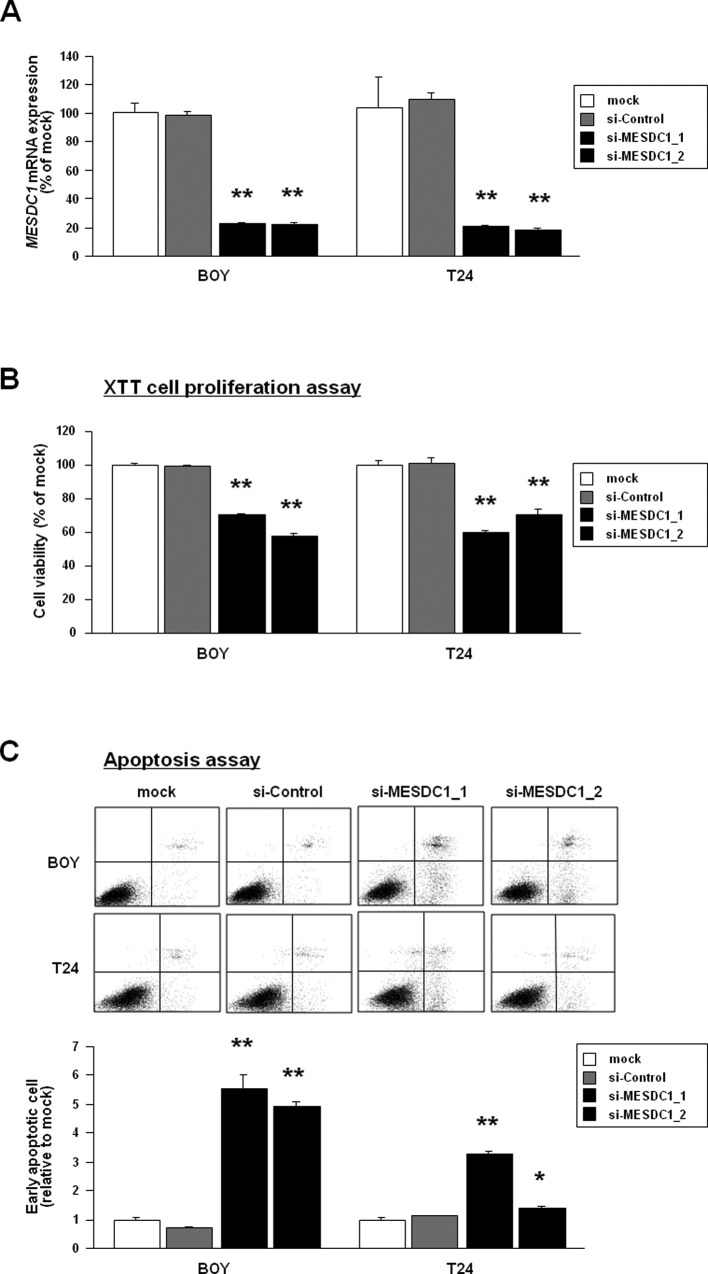

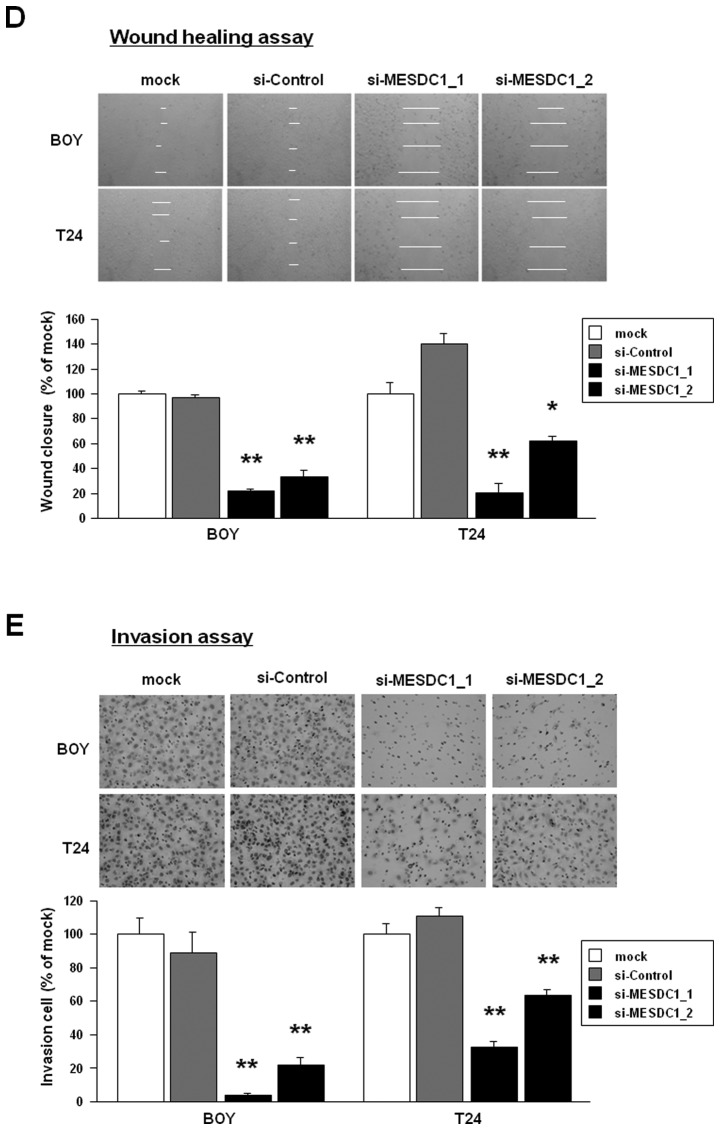
Effect of MESDC1 knockdown on BC cell viability revealed by siRNA transfection. (A), *MESDC1* mRNA expression in *miR-574-3p* transfectants (BOY and T24) compared with the controls. (B), XTT assays demonstrate significant cell growth inhibitions in si-MESDC1 transfectants in comparison with negative control siRNA-transfectants or mocks (untransfectants). ^**^P<0.0001. (C), Flow cytometric assays of apoptosis. Significant numbers of early apoptotic cells were observed in the si-MESDC1 transfectants. Early apoptotic cells can be seen in the bottom right quadrant. The histograms below show the percentages of early apoptotic cells out of the total measured cell population for si-control transfectants and si-MESDC1-transfectants. ^*^P<0.01. ^**^P<0.0001. (D), Wound healing assays demonstrated significant cell migration inhibition in the si-MESDC1 transfectants. Phase micrographs of the transfectants (BOY and T24) taken at 0 and 24 h after monolayer wounding are shown on the left. Quantitation of cell migration is shown on the right. ^*^P<0.01. ^**^P<0.0001. (E), Significant inhibition of cell invasion was observed in the si-MESDC1 transfectants. Phase contrast micrographs of invading transfectants (BOY and T24) are shown above. Quantitation of cell invasion is shown in the bottom panel (mean ± SEM of eight randomly selected ×200 magnification fields). ^**^P<0.0001.

To examine the functional role of MESDC1, we performed loss-of-function studies using two different si-MESDC1-transfected BC cell lines. The mRNA expression of *MESDC1* was markedly down-regulated by these si-MESDC1 transfections (Fig. 4A). The XTT assay demonstrated that cell proliferation was reduced to 71% and 57% of control by both siRNAs-treatment in BOY cells (P<0.0001) and that it was 60% and 70% of control (P<0.0001) in the T24 line (Fig. 4B). The early apoptotic cell fractions (right lower quadrant) were greater in the two si-MESDC1 transfectants than in the mocks and the si-control transfectants 72 h after transfection (relative to mock; BOY: 5.53±0.48, 4.93±0.15, 1.00±0.05, and 0.73±0.01, respectively, P<0.0001; T24: 3.30±0.08, 1.37±0.08, 1.00±0.05, and 1.11±0.03, P<0.0001 and P=0.0024) (Fig. 4C). These results suggested that knockdown of *MESDC1* expression might induce cell apoptosis in BC. The wound healing assay also demonstrated that wound closure ratio was reduced to 21% and 33% of mock by both siRNAs-treatment in BOY cells (each P<0.0001) and that it was 21% and 62% of control in the T24 cells (P<0.0001 and P=0.0007) (Fig. 4D). The matrigel invasion assay demonstrated that cell invasion ratio was reduced to 4% and 22% of mock by both siRNAs-treatment in BOY cells (each P<0.0001) and that it was 33% and 64% of control in the T24 cells (each P<0.0001) (Fig. 4E).

## Discussion

Since Gottardo *et al* reported in 2007 (8), several miRNA expression signatures of BC have been published (8–13). Several microRNAs signatures were found to have oncogenic or tumor suppressive function. However, it is difficult for investigators to choose the important miRNAs to be examined. We previously investigated chromosomal gain or loss regions in BC by using comparative genomic hybridization (CGH) arrays (18) and found a typical loss region on chromosome 4p which harbors tumor suppressive *miR*-*218* (17). In this study, we focused on *miR*-*574*-*3p* for two reasons: it was the seventh down-regulated miRNA in our miRNA signature specific to BC (16), and it was located on chromosome 4p. The expression levels of *miR*-*574*-*3p* were low in BC cell lines, and we found that it might have tumor suppressive function. Other tumor suppressive miRNAs in our previous study included *MiR-1*, *miR*-*133a*, and *miR-145*. Those miRNAs are located on chromosome 18q11.2 (*miR-1* and *miR-133a*) and 5q32 (*miR*-*145*) where typical chromosomal loss regions were identified in our previous CGH-array study (17). Thus, chromosomal loss regions might be one of the critical mechanisms regulating miRNA expression in tumor suppression.

In previous studies, serum *miR-574-3p* expression was found to be up-regulated in patients with hepatocellular carcinoma and liver cirrhosis (22), and tissue *miR-574-3p* expression was up-regulated in endometriomas (23). However, tissue *miR*-*574-3p* expression in various cancers had yet to be measured and previous studies did not address the functional role of *miR-574-3p*. In this study, we demonstrated that *miR*-*574-3p* significantly inhibited cell viability, migration, and invasion through its induction of cell apoptosis. In this study, there was no significant relationship between *miR*-*574-3p* expression and pathological parameters, possibly because our patient cohort was too small to evaluate the relationship between them. A future *in vivo* study would be helpful in clarifying the role of *miR-574-3p* in cancer development.

Our previous study demonstrated that tumor suppressive miRNAs, such as *miR-1*, *miR-133a*, and *miR-145* functioned through repression of *LIM* and *SH3 protein 1* (*LASP1*), *transgelin-2* (*TAGLN2*), and *Switch associated protein 70* (*SWAP70*) (15,16,24). These genes are commonly classified as actin-binding proteins. Actin-based structures are involved in cortical cell protrusions that mediate interactions between cells and the extracellular matrix (ECM), cell-to-cell interactions, cell migration, and cytoplasmic microfilamentous bundles that contribute to cell architecture and intracellular movement (25). It is plausible that the activation of these genes through ECM substrates contributes to tumor growth, migration, and invasion.

In this study, we focused on *MESDC1*, a potential target gene of *miR-574-3p*, because it was listed at the top of down-regulated genes in miRNA transfectants. *MESDC1* was actually up-regulated in BC cell lines. We demonstrated that *miR-574-3p* bound to the conserved site in the 3′ untranslated region of *MESDC1* mRNA in luciferase assays. We found several reports concerning MESDC1, but its functional role had not been investigated (26,27). In our functional studies using si-MESDC1 in BC cell lines, we demonstrated that cell viability, migration and invasion were all inhibited and apoptosis was induced. These results suggest that MESDC1 protein has an oncogenic function in BC and is regulated by *miR-574-3p* expression. In this study, there was no significant difference in *miR-574-3p* expression levels between clinical BCs and RNA obtained from the normal human bladders. Our cohort was too small to evaluate the difference between them. We demonstrated that oncogenic function was mediated by the *MESDC1* gene as well as other actin-binding proteins that we investigated previously. Our series of functional analyses of genes targeted by tumor suppressive miRNAs revealed that these actin-binding proteins might have anti-apoptotic effects in several cancer types (16,17,19,28–31). Further studies are necessary to elucidate the precise mechanisms by which apoptosis is induced by tumor suppressive miRNAs and knockdown of their target genes.

In conclusion, our results demonstrated that *miR-574-3p* was down-regulated in BC cell lines. We found decreased cell proliferation, migration, and invasive activities, and increased cell apoptosis in *miR-574-3p* transfectants, suggesting that *miR-574-3p* is a candidate tumor suppressive miRNA in human BC. Further, we found that *miR-574-3p* targeting of MESDC1 plays an important role in cell viability, migration, invasion, and apoptosis in BC cell lines.

## Figures and Tables

**Table I:** Down-regulated genes in *miR-574-3p* transfectants.

Entrez gene ID	Fold change (log2 ratio)				Gene name	Target sites
	Symbol	BOY	T24	Average		
59274	*MESDC1*	−2.01	−1.59	−1.80	*Mesoderm development candidate 1*	+
10950	*BTG3*	−1.88	−1.34	−1.61	*BTG family, member 3*	+
27430	*MAT2B*	−1.47	−1.41	−1.44	*Methionine adenosyltransferase II, beta*	+
11160	*ERLIN2*	−1.61	−1.15	−1.38	*ER lipid raft associated 2*	−
2021	*ENDOG*	−1.41	−1.28	−1.34	*Endonuclease G*	+
340508	*LOC340508*	−1.54	−1.09	−1.31	*Hypothetical protein LOC340508*	−
117178	*SSX2IP*	−1.37	−1.05	−1.21	*Synovial sarcoma, X breakpoint 2 interacting protein*	+
285761	*DCBLD1*	−1.40	−1.00	−1.20	*Discoidin, CUB and LCCL domain containing 1*	+
122416	*ANKRD9*	−1.19	−1.16	−1.17	*Ankyrin repeat domain 9*	+
3363	*HTR7*	−1.16	−1.03	−1.09	*5-hydroxytryptamine (serotonin) receptor 7 (adenylate cyclase-coupled)*	+
246329	*STAC3*	−1.01	−1.05	−1.03	*SH3 and cysteine rich domain 3*	−

## References

[1] Jemal A, Siegel R, Xu J, Ward E (2010). Cancer statistics, 2010. CA Cancer J Clin.

[2] Website of Center for Cancer Control and Information Services http://ganjoho.jp/professional/statistics/statistics.html.

[3] Herr H, Konety B, Stein J (2009). Optimizing outcomes at every stage of bladder cancer: do we practice it?. Urol Oncol.

[4] Zimmerman AL, Wu S (2011). MicroRNAs, cancer and cancer stem cells. Cancer Lett.

[5] Fendler A, Stephan C, Yousef GM, Jung K (2011). MicroRNAs as regulators of signal transduction in urological tumors. Clin Chem.

[6] Calin GA, Croce CM (2006). MicroRNA signatures in human cancers. Nat Rev Cancer.

[7] Esquela-Kerscher A, Slack FJ (2006). Oncomirs - microRNAs with a role in cancer. Nat Rev Cancer.

[8] Gottardo F, Liu CG, Ferracin M (2007). Micro-RNA profiling in kidney and bladder cancers. Urol Oncol.

[9] Yang H, Dinney CP, Ye Y (2008). Evaluation of genetic variants in microRNA-related genes and high risk of bladder cancer. Cancer Res.

[10] Ichimi T, Enokida H, Okuno Y (2009). Identification of novel microRNA targets based on microRNA signatures in bladder cancer. Int J Cancer.

[11] Catto JW, Miah S, Owen HC (2009). Distinct microRNA alterations characterize high- and low-grade bladder cancer. Cancer Res.

[12] Catto JW, Alcaraz A, Bjartell AS (2011). MicroRNA in prostate, bladder, and kidney cancer: a systematic review. Eur Urol.

[13] Han Y, Chen J, Zhao X (2011). MicroRNA expression signature of bladder cancer revealed by deep sequencing. PLoS One.

[14] Chiyomaru T, Enokida H, Tatarano S (2010). miR-145 and miR-133a function as tumor suppressors and directly regulate FSCN1 expression in bladder cancer. Br J Cancer.

[15] Chiyomaru T, Enokida H, Kawakami K (2010). Functional role of LASP1 in cell viability and its regulation by microRNAs in bladder cancer. Urol Oncol.

[16] Yoshino H, Chiyomaru T, Enokida H (2011). The tumour-suppressive function of miR-1 and miR-133a targeting TAGLN2 in bladder cancer. Br J Cancer.

[17] Tatarano S, Chiyomaru T, Kawakami K (2011). Mir-218 on the genomic loss region of chromosome 4p15.31 function as a tumor suppressor in bladder cancer. Int J Oncol.

[18] Matsuda R, Enokida H, Chiyomaru T (2011). LY6K is a novel molecular target in bladder cancer on basis of integrate genome-wide profiling. Br J Cancer.

[19] Yoshitomi T, Kawakami K, Enokida H (2011). Restoration of miR-517a expression induces cell apoptosis in bladder cancer cell lines. Oncol Rep.

[20] Takemoto M, Shirahama T, Miyauchi T (1997). Metanestin, a glycoprotein with metastasis-associated expression in transitional cell carcinoma of the urinary bladder. Int J Cancer.

[21] Sobin LH, Wittekind C (2002). TNM Classification of Malignant Tumors International Union Against Cancer (UICC).

[22] Gui J, Tian Y, Wen X (2011). Serum microRNA characterization identifies miR-885-5p as a potential marker for detecting liver pathologies. Clin Sci.

[23] Hawkins SM, Creighton CJ, Han DY (2011). Functional microRNA involved in endometriosis. Mol Endocrinol.

[24] Chiyomaru T, Tatarano S, Kawakami K (2011). SWAP70, actin-binding protein, function as an oncogene targeting tumor-suppressive miR-145 in prostate cancer. Prostate.

[25] Kureishy N, Sapountzi V, Prag S, Anilkumar N, Adams JC (2002). Fascins, and their roles in cell structure and function. Bioessays.

[26] Wines ME, Lee L, Katari MS (2001). Identification of mesoderm development (mesd) candidate genes by comparative mapping and genome sequence analysis. Genomics.

[27] Gingras AR, Bate N, Goult BT (2010). Central region of talin has a unique fold that binds vinculin and actin. J Biol Chem.

[28] Uchida Y, Chiyomaru T, Enokida H (2011). MiR-133a induces apoptosis through direct regulation of GSTP1 in bladder cancer cell lines. Urol Oncol.

[29] Nohata N, Hanazawa T, Kikkawa N (2011). Tumor suppressive microRNA-375 regulates oncogene AEG-1/MTDH in head and neck squamous cell carcinoma (HNSCC). J Hum Genet.

[30] Kawakami K, Enokida H, Chiyomaru T (2011). The functional significance of miR-1 and miR-133a in renal cell carcinoma. Eur J Cancer.

[31] Nohata N, Hanazawa T, Kikkawa N (2011). Identification of novel molecular targets regulated by tumor suppressive miR-1/miR-133a in maxillary sinus squamous cell carcinoma. Int J Oncol.

